# Açaí (*Euterpe olecareae* Martius) intake and its protective effect against 1,2-DMH-induced rat colon carcinogenesis

**DOI:** 10.1186/1753-6561-7-S2-P48

**Published:** 2013-04-04

**Authors:** Mariana F Fragoso, Luís F Barbisan

**Affiliations:** 1 Department of Pathology, Botucatu Medical School, UNESP, Botucatu, SP, Brazil; 2 Department of Morphology, Botucatu Biosciences Institute, UNESP, Botucatu, SP, Brazil

## Background

Açaí (*Euterpe olecareae* Martius) is an important crop consumed in Brazil that gained attention due to its potential antioxidant and anti-inflammatory properties. Various studies have shown that this exotic fruit may reduce the risk of many diseases, including cancer. The present study investigated the beneficial potential of açaí extract intake on the 1,2-dimethylhydrazine (DMH)-induced rat colon carcinogenesis.

## Materials and methods

Five groups were studied: G1 to G4 groups received four s.c. injections of DMH (40 mg/kg) twice a week during two weeks and G5 group received similar injections of EDTA (DMH vehicle). After two weeks of colon initiation, groups were fed basal diet (G1), basal diet containing 2.5% açaí extract (G2), basal diet containing 5.0% açaí extract (G3 and G5) or basal diet containing 0.2% of N-acetylcysteine (G4, positive control). Ten weeks after the beginning of the treatment, G1 to G4 groups were sacrificed for aberrant crypt foci (ACF) assay. Also, G1 and G3 groups were maintained on basal diet or basal diet containing 5.0% açaí extract, respectively, until week 20. Colons were analyzed for ACF development (week 10) or tumor incidence, multiplicity and histological analyses (week 20).

## Results

The number of total aberrant crypts (AC) was significantly reduced in G3 and G4 groups when compared to G1 group (*p*=*0.036*). Data from ACF (1-3 AC) was significantly lower in G3 and G4 groups (*p*=*0.011* and *p*=*0.037*, respectively). In the colon tumor assay, G1 group developed invasive (75%) and noninvasive (25%) tumors whereas G3 group developed only noninvasive tumors (100%).

## Conclusions

These results suggest that açaí extract intake is potentially beneficial for early reduction of the number of AC and may affect tumor development and late malignancy in male Wistar rats initiated for colon carcinogenesis.

## Financial support

CAPES.

